# Stricture of the Esophagus

**Published:** 1872-09

**Authors:** James S. Bailey

**Affiliations:** Albany, N. Y.


ART. II.—*Stricture of the Esophagus.* BY JAMES S. BAILEY, M. D., Albany, N. Y.

Two years ago a lad ten years of age was presented at my office for examination. He was a pale emaciated looking boy with no symptoms of disease, except he could not swallow food and seemed to be sinking from exhaustion.

The father gave me the following history: He was engaged in a petroleum refinery, and for this purpose they used a concentrated form of potash. He usually took with him his dinner also a small pot of coffee.

The boy upon his fathers return at night was in the habit of drinking the coffee if any was left. Instead of bringing home an empty coffee pot one night, he put in it some of this caustic potash for the purpose of removing some grease spots from the floor.

The boy, as was his habit, turned the pot to his mouth, but instead of finding coffee he took a mouthful of the potash, and instead of spitting it out he swallowed it, which excoriated the mouth as well as the esophagus.

A physician was sent for who refused to come, and said nothing could be done. After about one month the inflammatory symptoms had subsided, but he found great difficulty in swallowing even liquids. In taking a draught of water he would close his mouth and in the act of forcing it down a portion would escape through the nose.

A physician now for the first time was consulted who prescribed carbolic acid gargle, this was continued two weeks without relief, when the discouraged father brought him to me, a distance of nearly two miles, which the child walked.

After hearing the history I did not make an examination, but ordered him carried home and promised to call at his house next day, believing that I had to deal with a strictured esophagus.

Upon my first visit I entirely failed to penetrate beyond the strictured portion which was about an inch beyond the cricoid cartilage.

The next day I succeeded in passing through the stricture a very small bougie, which caused a free discharge of glairy mucus. I continued to introduce the instrument from day to day and at the expiration of one month could introduce a size no larger than a No. twelve bougie.

His swallowing was improved after the first introduction and he then partook freely of fluid nourishment of beef tea, milk &c., which caused rapid improvement in flesh; it seemed as if the little fellow could never satisfy his craving for food.

The orifice of the stricture seemed cartilaginous. He was finally enabled to take semi-solid food and meats cut very fine.

I now requested my patient to visit my office which he failed to do as he now could be sufficiently nourished, probably fearing he would be called upon to pay a portion of his indebtedness. I soon after lost track of my patient by his removal until one year ago, when I accidentally came across him and learned that he still experienced trouble in deglutition and could not swallow as well as when I last saw him, but could take enough food to sustain life.

This case was one of rare interest and I believe one that seldom occurs. Timely efforts only saved the boy from starvation. There was frequently considerable spasmodic muscular contraction of the esophagus upon attemptiug to remove the bougie, but repeated introductions caused this to gradually disappear.

It is remarkable that the patient could take sufficient nourishment to sustain life, though this small opening and accumulate flesh so rapidly.

I am now enabled to report the lad in vigorous health though still suffering considerable embarrassment in deglutition.

